# Reviews and Book Notices

**Published:** 1883-11

**Authors:** 


### Reviews and Book Notices.

---

ARTICLE X.—DISEASES OF WOMEN. A Manual for Students and Practitioners. By ARTHUR M. EDIS, M.D., Lond., F.R.C.P., M.R.C.S., Assistant Obstetric Physician to the Middlesex Hospital, Consulting Obstetric Physician to the City Provident Dispensary, etc., etc.

Few, if any, manuals on the subject of gynæcology before the profession are written in as clear, concise and comprehensive a manner as this one. It contains thousands of excellent hints as to care and details in treatment of patients, which will prove of the greatest value to the young practitioner.

The author gives considerable prominence to uterine displacements and their treatment by mechanical means, although he cautions the practitioner against placing too much reliance upon mechanical appliances.

The differential diagnosis of abdominal tumors is tabulated, as far as practicable, for the convenience of the busy practitioner. and is given in the most exhaustive manner. E. M. N.

---

ARTICLE XI.—TRANSACTIONS OF THE AMERICAN GYNÆCOLOGICAL SOCIETY FOR 1880.

Volume 5 is before the public. This is a neatly bound volume containing 450 pages.

The papers were prepared and read by some of the most distinguished men of the medical profession of the United States and the discussions were most able. Among the papers read which were of more than ordinary interest, may be mentioned: "What is the Proper Field for Battey's Operation," by Dr. Robert Battey; "Extripation of an Encephaloid Kidney," by Dr. W. H. Byford, and "Posture in Labor—An Ethnological Study," by Dr. G. J. Engelmauer. E. M. N.

---

ARTICLE XII.—A CLINICAL HAND-BOOK ON THE DISEASES OF WOMEN. By U. SYMONGTON BROWN, M.D., Member of the Gynæcological Society of Boston, Fellow of the Mass. Medical Society, etc.

The author does not claim that this book is a treatise but acknowledges omitting all that would not be of much practical use to the student and practitioner. Certainly the most noticable feature of the book is its incompleteness. The author divides the subject in the usual order and gives the most salient points in a very clear and readable manner. E. M. N.

Article XIII.—The Grounds of a Homœpath's Faith. By Samuel A. Jones, m.d, Professor of Materia Medica and Therapeutics in the Homœopathic Medical College, the University of Michigan.

This little book consists of a series of three lectures delivered before the medical matriculants of the University of Michigan. The first lecture is entitled "The Law of Similars," but for the greater part is a sentimental sketch of the life of "the faith-filled man," Samuel Hahnemann. He differentiates this school of medicine in the following words: "If I were asked to state what chiefly distinguishes the homœopathic physician from his older brother in the science and art of medicine, I should at once reply: Not the law of cure, not the infinitesimal dose, not the Hahnemannian hypothesis of chronic diseases; none of these, but simply this—his fixed faith in the efficiency of drugs."

The topic of the second lecture was: "The Single Remedy a Necessity." He speaks of the aid chemistry has been in medicine as: "A tale for the medical 'marines,' an apple of Sodom, a mirage, a humbug," and adds: "Chemistry can no more foretell the physiological action of a compound than I can imagine what the Dean of the 'Department of Medicine and Surgery' wonld say if he woke up some morning and found himself in bed with a homœopath."

The third and last lecture, "The Minimum Dose," abounds with logic as profound as the two previous lectures, and closes with a touching appeal for sympathy because of the persecution homœopathy had received. A physician might make this book quite valuable to him by lending it to his friends who have some regard for homœopathy; it would cure them. E. M. N.

---

Article XIV.—On the Use of the Cold Pack followed by Massage in the Treatment of Anæmia. By Mary Putnam Jacobi, m.d., and Victoria A. White, m.d.

The writer gives the clinical histories, with a consideration of the results, of eleven anæmic patents treated as the title of the book would indicate: a cold pack from ten minutes to an hour's duration, followed by massage for one hour. This treatment was used from the belief that it would increase the stupidity of tissue metamorphoses, thus promoting assimilation. Carefully prepared tables were made, showing the comparative amount of urea, urine and inorganics eliminated during the treatment, with the amount eliminated during the hours between treatments. Nine of the cases, who received this treatment every day for six weeks, were greatly benefited, while two, who were nevertheless under the same treatment, remained unimproved. The book is a very readable one. E. M. N.

---

ARTICLE XIV.—WHAT TO DO FIRST IN ACCIDENTS AND EMERGENCIES. By Chas. W. Dulles, M.D. Second edition, revised and enlarged.

This beautiful little book has not lost its attractiveness in its revision and second edition. Of handy size for the pocket or satchel, its companionship is of value to the traveler or mechanic in his workshop. Accidents and emergencies most liable to occur in common life are suggested, and simple, practical direction given for the first proceeding; not to take the place of calling the physician or surgeon, but to fill up with timely action the fateful moments before the skilled assistant arrives. The matter is arranged in most convenient form, well indexed and preceded by a table of contents.

